# Neuroplasticity after upper-extremity rehabilitation therapy with sensory stimulation in chronic stroke survivors

**DOI:** 10.1093/braincomms/fcac191

**Published:** 2022-07-24

**Authors:** Christian Schranz, Amanda Vatinno, Viswanathan Ramakrishnan, Na Jin Seo

**Affiliations:** Department of Health Sciences and Research, Medical University of South Carolina, Charleston, SC 29425, USA; Department of Health Sciences and Research, Medical University of South Carolina, Charleston, SC 29425, USA; Department of Public Health Sciences, Medical University of South Carolina, Charleston, SC 29425, USA; Department of Health Sciences and Research, Medical University of South Carolina, Charleston, SC 29425, USA; Department of Rehabilitation Sciences, Medical University of South Carolina, Charleston, SC 29425, USA; Ralph H. Johnson VA Medical Center, Charleston, SC 29401, USA

**Keywords:** EEG, coherence, ERSP, vibratory stimulation, upper extremity

## Abstract

This study investigated the effect of using subthreshold vibration as a peripheral sensory stimulation during therapy on cortical activity. Secondary analysis of a pilot triple-blinded randomized controlled trial. Twelve chronic stroke survivors underwent 2-week upper-extremity task-practice therapy. Half received subthreshold vibratory stimulation on their paretic wrist (treatment group) and the other half did not (control). EEG connectivity and event-related de-/resynchronization for the sensorimotor network during hand grip were examined at pre-intervention, post-intervention and follow-up. Statistically significant group by time interactions were observed for both connectivity and event-related spectral perturbation. For the treatment group, connectivity increased at post-intervention and decreased at follow-up. Event-related desynchronization decreased and event-related resynchronization increased at post-intervention, which was maintained at follow-up. The control group had the opposite trend for connectivity and no change in event-related spectral perturbation. The stimulation altered cortical sensorimotor activity. The findings complement the clinical results of the trial in which the treatment group significantly improved gross manual dexterity while the control group did not. Increased connectivity in the treatment group may indicate neuroplasticity for motor learning, while reduced event-related desynchronization and increased event-related resynchronization may indicate lessened effort for grip and improved inhibitory control. EEG may improve understanding of neural processes underlying motor recovery.

## Teaser

Twelve stroke survivors underwent 2-week upper-extremity task-practice therapy while receiving either subthreshold vibratory stimulation (treatment group) or no stimulation (control). EEG connectivity increased and event-related desynchronization decreased for the treatment group which may represent motor learning and improved motor function from therapy supplemented with the vibratory stimulation.

## Graphical Abstract

**Graphical Abstract:**
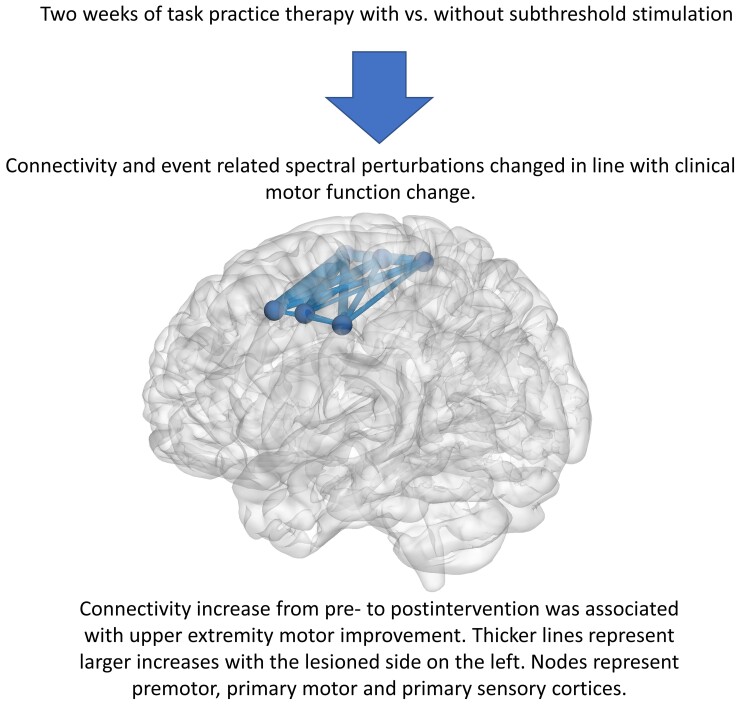

## Introduction

Stroke is a leading cause of long-term disability worldwide.^1^ Motor impairment post-stroke limits stroke survivors’ abilities for activities of daily living.^2^ Many stroke survivors do not fully recover their upper limb function even after completion of standard-of-care rehabilitation treatment.^3^ One method to improve rehabilitation outcome is to augment therapy by adding peripheral sensory stimulation.^4^ Meta-analysis shows that the use of peripheral sensory stimulation in conjunction with standard upper limb rehabilitation therapy improves motor function more than therapy alone in chronic stroke survivors.^4^

The proposed neural mechanism is as follows. Sensory input is a powerful driver of change in the motor cortex^5–7–8^ Peripheral sensory stimulation stimulates not only the sensory afferent pathway but also the motor pathway via direct projections from the sensory cortex to the motor cortex.^9–13^ Ample evidence exists for single-session effects of peripheral sensory stimulation on activity and excitability of the sensorimotor cortex as well as corticomuscular connectivity. As for multi-session effects of peripheral sensory stimulation, only two studies exist. One study found that transcutaneous electrical nerve stimulation for 40 min before upper-extremity therapy twice a week for 4 weeks increased corticomuscular coherence between the primary motor cortex and thenar eminence, compared with therapy alone, in chronic stroke survivors.^14^ The other study investigated muscle vibration on the flexor carpi radialis and biceps brachii for 10 min before physiotherapy for three sessions compared with physiotherapy only, in chronic stroke survivors. The group receiving the muscle vibration showed a decreased resting motor threshold, increased motor map volume and increased short interval intracortical inhibition for stimulated muscles using Transcranial Magnetic Stimulation.^15^ These previous studies indicate that peripheral sensory stimulation induces motor pathway neuroplasticity.^5,6^

The present study examined the multi-session effect of a recently developed subthreshold random-frequency vibratory stimulation applied to the wrist.^16,17^ This stimulation has been developed based on the following theoretical framework. Subthreshold vibration can activate mechanoreceptors in the wrist skin and corresponding sensory afferents to the central nervous system.^18–20^ Our previous results showing changes in cortical activation during this stimulation using EEG support this notion.^21,22^ These afferent signals add small random currents to neurons in the sensorimotor cortex, which can trigger coherent^23,24^ firing^25^ at the peak of input related to hand tasks, thus consequently enhancing signal transmission and neural communication.^26–29^ The enhanced neural communication may contribute to improved motor recovery.^30,31^

Indeed, a previous pilot triple-blind randomized controlled trial using this stimulation during upper-extremity task-practice therapy showed significantly greater improvement in gross manual dexterity as assessed by the Box and Block Test, compared with the group that did not receive the stimulation during therapy.^30^ However, whether using the stimulation during therapy indeed enhances neural communication over multiple sessions has not been examined. Neural communication can be assessed using EEG connectivity.^26,27,32,33^ Therefore, we hypothesized that the use of subthreshold random-frequency vibratory stimulation would result in increased cortical sensorimotor network connectivity.

## Materials and methods

### Design

This study is a secondary analysis of the triple-blinded (patients, therapists and assessors) pilot randomized controlled trial.^30^ Twelve chronic stroke survivors wore a small vibrator on the paretic wrist and underwent task-practice therapy for 2 h three times a week for 2 weeks. They were randomized to either a treatment group that received subthreshold random-frequency vibration at 60% of the sensory threshold or a control group that received no vibration from the vibrator during therapy (*n* = 6/group)^30^ through a block randomization. Upper-extremity motor function using the Box and Block Test and EEG was assessed at pre-intervention, post-intervention (on average 6 days after therapy completion) and follow-up (on average 19 days after therapy completion).

### Participants

Inclusion criteria were adults, at least 6 months post-stroke with mild to moderate impairment based on the Fugl-Meyer Upper-Extremity Assessment score.^34^ Exclusion criteria were not being able to follow instructions, having received botulinum toxin injection within the 3 months before or during enrolment, and receiving concurrent upper-extremity therapy. The demographic characteristics were comparable in the two groups.^30^ Participants were on average 63 years of age [standard deviation (SD) = 8], seven males and five females, and had the average time since the stroke of 5 years (SD = 5). One participant in the control group did not complete the follow-up EEG assessment. This study was approved by the local Institutional Review Board and informed consent was obtained from all participants.

### EEG acquisition

EEG was recorded using a 96-channel actiCAP, BrainAMP MR plus amplifier and BrainVision Recorder software (BrainVision LLC, Morrisville, NC, USA). Electrodes were positioned using the 10–20 system with a ground at AFz and a reference at FCz. The signal was applied with a bandwidth filter at 0.1–200 Hz and a notch filter at 60 Hz and recorded at 1 kHz.

The EEG protocol used in a previous study^21^ was followed. Specifically, participants were seated in front of a computer screen, with the forearm resting on an armrest and the hand on force sensors (Mini40, ATI Industrial Automation Inc., Apex, NC, USA). They were instructed to grip the sensors using the thumb and index finger of the paretic hand upon a visual cue generated by a custom LabVIEW programme (National Instruments, Austin, TX, USA). They practiced gripping at 4 N using visual feedback before data collection. During the EEG data collection, each grip cue lasted 2 s long, followed by a 5–6 s rest cue. Each participant completed 100 grip trials.

### MRI acquisition

To enable source localization of EEG activity, structural T_1_-weighted brain MRI was obtained. Source EEG data were used instead of channel data because EEG channel data do not directly relate to the underlying source activity and channel data also suffer from spurious estimation of connectivity due to the effects of field spread and volume conduction.^35,36^ Brain MRI with an isometric 1 mm^3^ voxel size was obtained via the MPRAGE sequence^37^ using a Siemens 3 T TIM Trio MRI scanner (Siemens AG, Munich) for 10 participants. The other two participants had contraindications to MRI.

**Figure 1:**
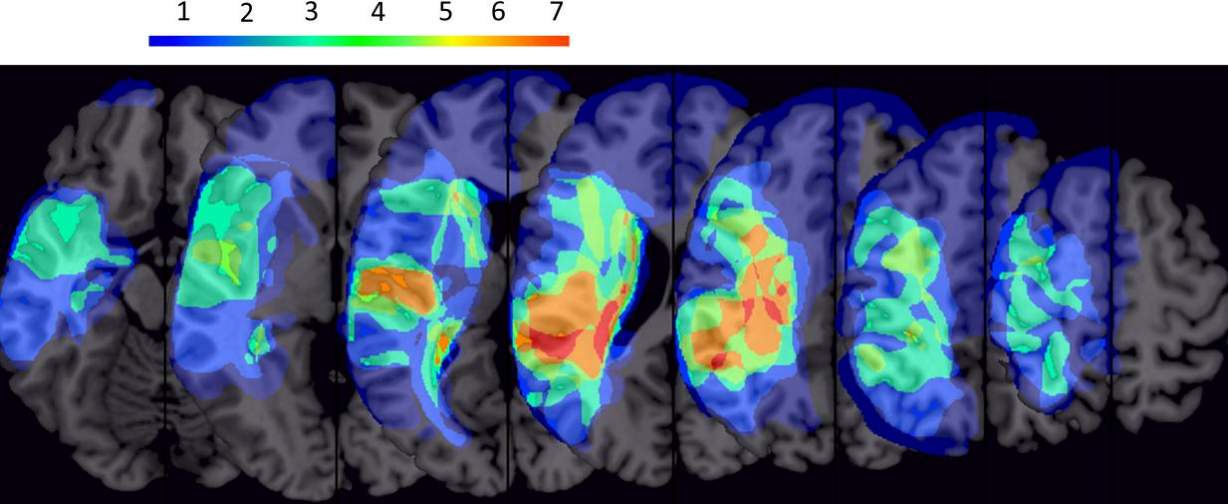
**Lesion locations for the participants**. The colour bar indicates the number of participants with a lesion at each location.

The lesion characteristics of the participants are visualized in Fig. 1. Specifically, the lesion locations for the 10 participants were manually drawn in MRIcron^38^ and normalized into the standard Montreal Neurological Institute (MNI) space.^39^ Participants had lesions in the central region most frequently.

### EEG analysis

EEG data were preprocessed using EEGLAB toolbox^40^ in MATLAB (The MathWorks, Inc., Natick, MA, USA). EEG data were resampled at 500 Hz. Bad channels were replaced using a spherical interpolation.^41^ Data were re-referenced using an average reference. Artefacts were eliminated using artefact subspace reconstruction.^42^ Segments with noisy EEG data or no grip were identified from visual inspection of the EEG and force sensor data, respectively, and excluded from further analysis. After this exclusion, an average of 87 grip trials (SD = 18) remained per participant. The data were divided into epochs ranging from −2 to 4.5 s relative to the grip cue onset.

Using MRI data, the cortical surface was reconstructed and the brain regions were segmented in Freesurfer.^43^ The segmented brain was imported in Brainstorm^44^ and registered with the nasion, right/left auricular points, inion, midline and anterior/posterior commissure. The segmentation for Desikan-Killiany atlas^45^ was visually checked. Incorrect segmentation was found for five participants because of large lesions. Thus, their regions of interest were manually drawn. For the two participants with contraindications to MRI, the MNI average brain^39^ was used. A custom head model was created for each participant’s brain using the Symmetric Boundary Element method.^46^ Cortical source activity was computed using minimum norm estimation.^47^

Corticocortical connectivity was computed for the sensorimotor network. Corticocortical connectivity is the correlation between signals in different brain areas in the frequency domain.^32^ Connectivity was estimated using the imaginary coherence to avoid volume conduction artefact and examine true brain interaction.^48^ The regions of interest included premotor, primary motor and primary sensory cortices of both ipsilesional and contralesional hemispheres. Those regions were selected because premotor and primary motor cortices are the main centres for planning and executing movements^49^ based on the processing and translation of sensory input in the primary sensory cortex.^50^ Both hemispheres were examined because both hemispheres are engaged for fine motor tasks such as precision grip in healthy individuals.^51,52^ Connectivity for two phases of grip was examined. Specifically, connectivity during the 1 s period immediately before and after the grip cue onset represented connectivity for grip preparation and initiation, respectively. These two phases were examined because cortical activity during motor preparation and initiation has been shown to be most relevant for motor performance.^53,54^ Beta frequency band between 13 and 29 Hz was used because this frequency is known to be used for long-range corticocortical communication, which is relevant for the neural communication in the cortical sensorimotor network targeted by the subthreshold random-frequency vibratory stimulation.^55^

In addition, for secondary analyses, event-related spectral perturbation (ERSP) was also assessed in order to investigate the change in local brain activation in the sensorimotor cortices.^56–59^ Time-frequency analysis was performed using Morlet wavelets. The same regions of interest as for connectivity were examined. ERSP was computed as % power change from the baseline. Event-related desynchronization (ERD) for grip initiation and grip termination was determined as the minimum ERSP during the 2 s grip cue and 2 s period after the grip cue changed to the rest cue, respectively. Event-related resynchronization (ERS) after grip was determined as the maximum ERSP during the 2 s period after the grip cue changed to the rest cue. While both alpha and beta frequency bands have been shown to be relevant for sensorimotor processing.^56,60–65^ The subthreshold random-frequency vibratory stimulation was shown to induce larger alpha ERD change compared with beta,^21^ indicating that the stimulation led to arousal, release of inhibition and increase in supporting network activity.^63–65^ Thus, alpha ERSP (8–12 Hz) was examined.

### Statistical analysis

Linear mixed model analyses were performed using SAS (SAS Institute Inc., Cary, NC, USA). The factors included in the model for connectivity were group (treatment or control), time (pre-intervention, post-intervention and follow-up), brain region pair (among premotor, primary motor and primary sensory cortices of the ipsilesional and contralesional hemispheres) and grip phase (grip preparation and initiation). For ERSP, the model included group, time, brain region and grip phase (grip initiation, grip termination ERD, post-grip ERS). Diagnostics were performed to verify assumptions and to choose the appropriate structure for the within-subject correlations over time. An autoregressive [AR(1)] structure was used for the correlations. As suggested by the diagnostics, connectivity and ERSP data were transformed to inverse and square root, respectively, to achieve normality. The significance level of 0.05 was used. Linear contrasts were estimated when interactions were significant to further examine which pairwise comparisons led to statistical significance. Tukey *post hoc* adjustments were made.

## Results

### Corticocortical connectivity

**Figure 2:**
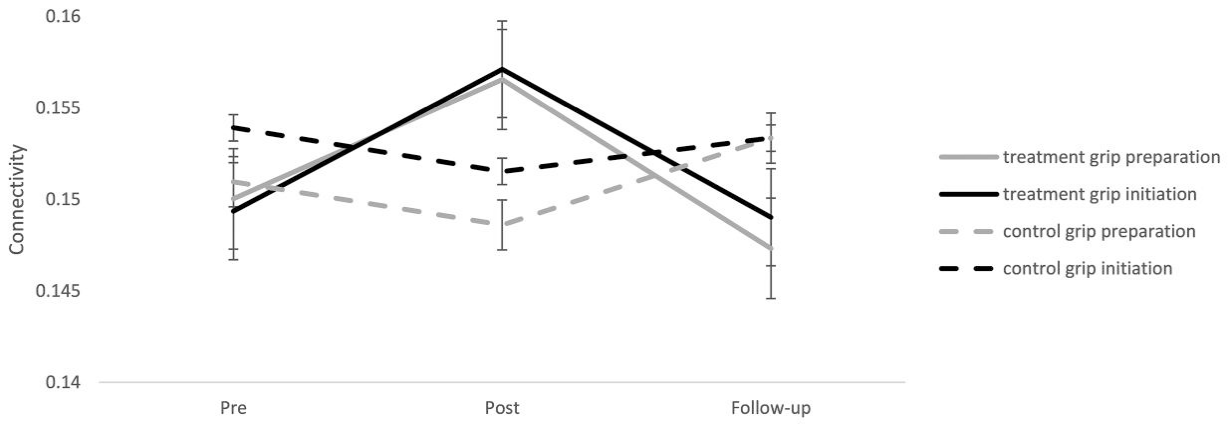
**Connectivity changes: connectivity during the two grip phases for the treatment and control group over pre, post and follow-up time points**. The average data for the regions of interest are shown. Error bars show standard error. Connectivity changed differently for each group [group × time, *F*(1,2) = 95.51, *P* < 0.001] based on linear mixed model analysis.

Connectivity changed over the three time points differently by group (group × time *P* < 0.001, Fig. 2). For the treatment group, connectivity increased at post-intervention (*P* < 0.001) and decreased at follow-up compared with pre-intervention (*P* < 0.001). For the control group, connectivity decreased at post-intervention (*P* < 0.001) and returned to the pre-intervention level at follow-up (*P* = 0.179). At pre-intervention, groups did not differ (*P* = 0.933). However, at post-intervention, the treatment group had higher connectivity than control (*P* = 0.005). This difference did not persist to follow-up (*P* = 0.483). Group by time by phase interaction was also found to be significant (*P* = 0.030, Fig. 2). There was no significant effect for the brain region pair or its interactions. Individual connectivity changes are presented in Supplementary Figure 1.

### Event-related spectral perturbation

**Figure 3:**
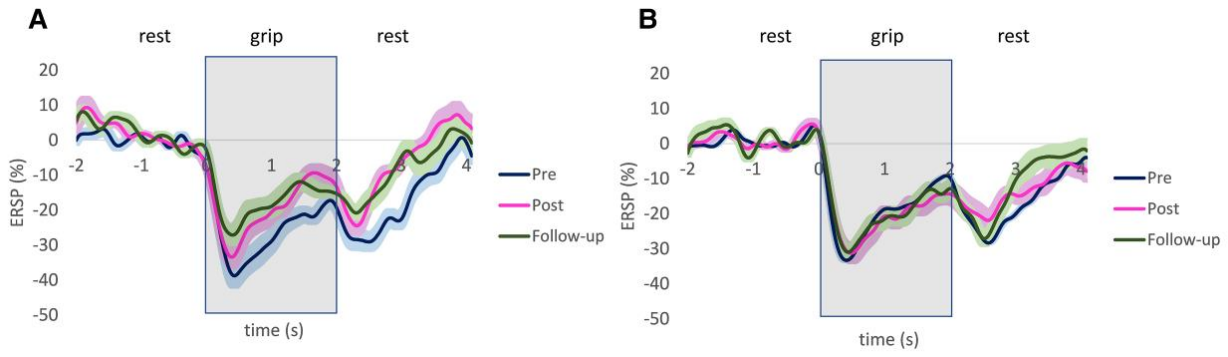
**ERSP changes**. ERSP for pre, post and follow-up for the treatment (**A**) and control group (**B**). The shaded areas represent standard error. The time when the grip cue was presented is marked with a grey rectangle. ERSP changed differently for each group [group × time, *F*(1,2) = 16,77, *P* < 0.001] based on linear mixed model analysis.

ERSP changed over the three time points differently by group (group × time *P* < 0.001, Fig. 3). *Post hoc* tests showed that the treatment group had reduced ERD for both grip initiation and termination and increased ERS from pre- to post-intervention (*P* = 0.016, *P* = 0.006, *P* < 0.001, respectively) and these changes were largely maintained at follow-up (*P* < 0.001, *P* = 0.004, *P* = 0.059, respectively, compared with pre). There were no significant changes for ERD and ERS for the control group. Time by group by phase interaction was also significant (*P* = 0.014, Fig. 3). The brain region and its interactions were not found to be significant. Individual changes for ERD during grip initiation, ERD during grip termination, and ERS are presented in Supplementary Figures 2, 3, and 4, respectively.

## Discussion

This study showed that the application of subthreshold random-frequency vibratory stimulation during upper-extremity therapy modified cortical sensorimotor activity differently than therapy alone in stroke survivors. These EEG findings complement the previously published clinical finding of the same cohort that showed improvement in gross manual dexterity for the treatment group, compared with no change for the control group.^30^ Importantly, the changes in connectivity and ERSP measured by EEG were associated with the change in the clinical upper-extremity motor function score.^30^ Therefore, this study provides support for EEG biomarkers for motor recovery.^58,66^ The different patterns of neuroplasticity that are associated with different clinical outcomes are discussed in detail below.

### Corticocortical connectivity

The stimulation used in this study was designed to enhance neural communication in the cortical sensorimotor network.^30^ Neural communication was assessed using corticocortical connectivity.^26,27^ It was found that the stimulation indeed led to increased cortical sensorimotor network connectivity in the treatment group from pre- to post-intervention compared with the control group. Therefore, the stimulation appears to enhance neural communication in the cortical sensorimotor network as seen by the increased connectivity, which may underlie the motor improvement seen in the treatment group.

Change in corticocortical connectivity is also regarded as a robust biomarker for motor improvement.^66^ Immediately after stroke, connectivity is typically reduced compared with individuals without stroke.^67,68^ With recovery, connectivity progressively increases, even in the chronic phase.^67,68^ Furthermore, connectivity has been shown to be proportionally modulated with sensorimotor learning.^69,70^ Therefore, an increase in connectivity may indicate motor learning necessary for motor recovery.^66,71^ Accordingly, our study showed that the treatment group that increased connectivity at post-intervention improved their motor function, while the control group that did not increase connectivity did not improve motor function.

Interestingly, at follow-up, motor improvement was sustained,^30^ but connectivity increase was not, in the treatment group. It has been shown that the brain connectivity, excitability or motor map volume changes during motor skill learning, but not later.^71–74^ It may be because with motor learning, connections among distant brain regions become more direct as evidenced by shorter path length with increased efficiency.^75^ Thus, the follow-up time point with regressed connectivity and retained motor improvement may represent the state after the new motor skills were learned and consolidated.

### Event-related spectral perturbation

ERD in the sensorimotor cortex reflects the extent to which the cortical pyramidal cells are activated during sensorimotor tasks^76^ with the hand.^77,78^ In stroke survivors, an increase in ERD in the sensorimotor cortex has been associated with an increased need for concentration and excitatory drive of pyramidal cells for the task.^77^ Therefore, the reduced ERD for the hand grip task in the treatment group at post-intervention and follow-up may indicate the lessened need for cortical engagement and effort^59^ required to perform the grip task as a result of motor improvement.^57^ This trend coincides with the clinical motor improvement seen in the treatment group at both post-intervention and follow-up.^30^

The treatment group also showed an increased ERS immediately after the grip. ERS rebound after the completion of a motor task reflects inhibitory control of the motor cortex^79^ as well as processing of somatosensory afferents post-movement as it also occurs after passive movement.^80^ Peripheral sensory stimulation has previously been shown to increase ERS over somatosensory cortex after movement and improve inhibitory control.^15,81^ Therefore, the increase in ERS for the treatment group in this study may reflect increased processing of afferent inputs and inhibitory control.

### Brain regions

Changes in connectivity and ERSP did not differ among the regions of interest in the ipsilesional and contralesional hemispheres, since there was no significant interaction involving the brain region. This finding was anticipated within the ipsilesional hemisphere since the regions of interest are the nodes of the sensorimotor network and thus intervention-induced changes should occur across all regions of interest in the ipsilesional hemisphere. As for the contralesional and interhemispheric connectivity, there exists a common view that contralesional activity represents compensation in lieu of the ipsilesional activity.^82,83^ However, brain activity is not always lateralized: while gross motor tasks such as power grip are associated predominantly with contralateral brain activity, fine motor tasks such as precision pinch used in the present study require bilateral brain activity in healthy adults.^52^ Such bilateral brain activity for fine motor tasks has been repeatedly demonstrated in the literature in both stroke survivors and healthy adults.^21,63,84^ Furthermore, improvements in hand motor control have been shown to be associated with changes in interhemispheric coherence in stroke survivors.^51^ Therefore, it is surmised that bilateral brain activity with intra- and interhemispheric communication is essential normal brain function needed for fine motor tasks and thus, our results showing treatment-induced changes in the bilateral sensorimotor network may be the normal course of neuroplasticity for fine motor improvements.

## Limitation

Limitations of this study include the small sample size and short study duration. Although we were able to show the differences in corticortical connectivity and ERSP between the two groups in this pilot study, a future study may employ a larger study sample and a longer treatment duration as well as a longer follow-up duration.^85^ The future larger study may also investigate the influence of other factors on recovery and neural plasticity including sex as a biological variable and lesion size. In addition, this study did not capture EEG channel positions for individual participants. Thus, actual EEG channel locations may have been slightly different from the standard location used in the source modelling.

## Future direction

Our findings encourage a possibility that interventions can be designed to target neuroplasticity such as the cortical sensorimotor network connectivity for motor improvement. Improved understanding of neuroplasticity underlying post-stroke motor recovery may spark the development of new interventions directly targeting the relevant brain dynamics and thus result in more effective rehabilitation treatment of motor impairment in persons with stroke.

## Conclusion

This study found a different pattern of neuroplasticity by adding subthreshold random-frequency vibratory stimulation to the upper-extremity task-practice therapy, compared with therapy only without stimulation. This different pattern of neuroplasticity was associated with the different pattern of motor improvement seen between the groups.^30^ As hypothesized, the addition of the stimulation resulted in increased sensorimotor network connectivity at post-intervention (on average 6 days after completion of 2-week therapy) in the treatment group, compared with the control group. At follow-up (on average 19 days after completion of 2-week therapy), the connectivity regressed towards pre-intervention. Decreased ERD and increased ERS were also found in the treatment group at post-intervention which were sustained at follow-up. In light of the greater improvement in gross manual dexterity seen in the treatment group compared with the control group in this cohort,^30^ these changes in the connectivity, ERD and ERS are interpreted as motor learning, a consequent reduction in the required effort and more efficient processing of sensory afferents as well as inhibitory control, respectively. These results support the promise of EEG as a biomarker for motor learning and recovery.^66,71^ Understanding the pattern of neuroplasticity underlying motor recovery may inspire the development of novel interventions directly targeting the recovery mechanisms.

## Supplementary Material

fcac191_Supplementary_Data

## Data Availability

The data that support the findings of this study are available upon request to the corresponding author.

## Abbreviations

ERD =: event-related desynchronization
ERS =: event-related resynchronization
ERSP =: event-related spectral perturbation
MNI =: Montreal Neurological Institute
