# Supplementary material for: Neuroplasticity after upper-extremity rehabilitation therapy with sensory stimulation in chronic stroke survivors
Source: Brain Commun. 2022 Jul 24;4(4):fcac191. doi: 10.1093/braincomms/fcac191 (PMC9351980; doi:10.1093/braincomms/fcac191)
Supplement: fcac191_Supplementary_Data [file fcac191_supplementary_data.pdf]

Supplemental Materials:

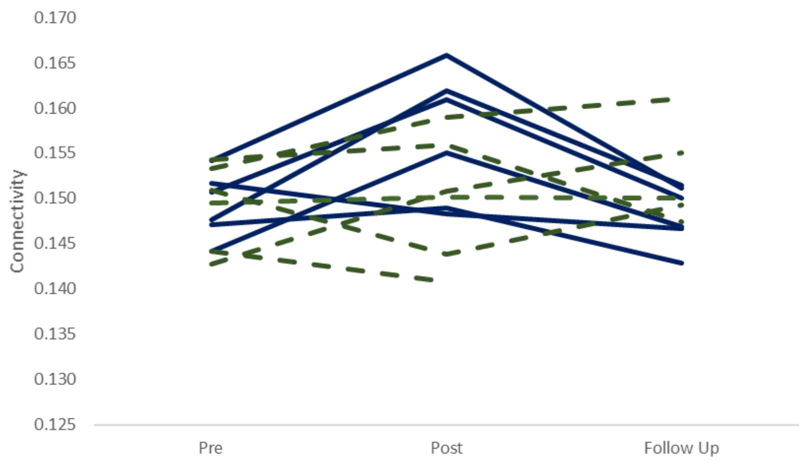

Supplementary Figure 1: Individual data for connectivity from pre-intervention to post-intervention to follow up for the treatment group participants (solid lines) and the control group participants (segmented lines)

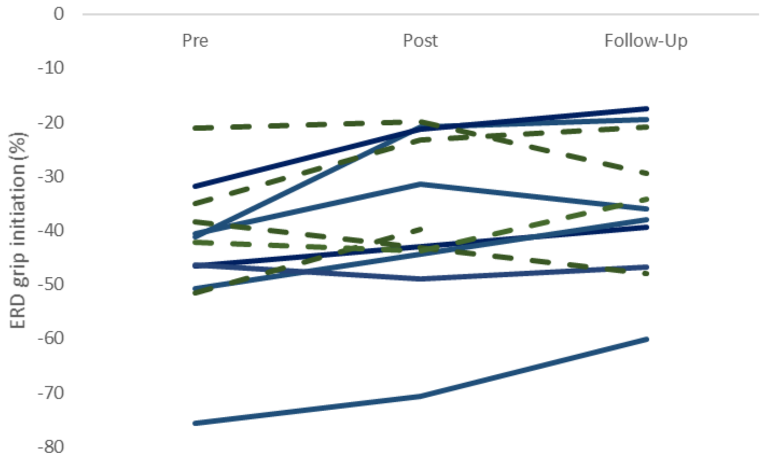

Supplementary Figure 2: Individual data points for both groups for event related desynchronization during grip initiation from pre-intervention to post-intervention to follow up for the treatment group participants (solid lines) and the control group participants (segmented lines)

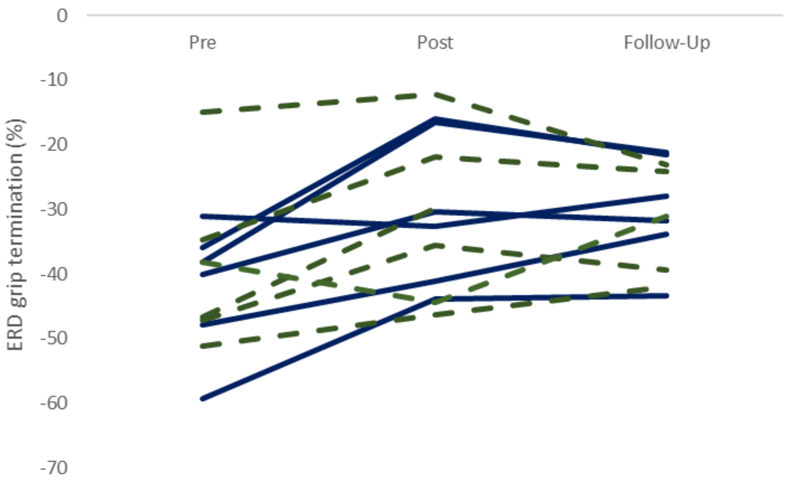

Supplementary Figure 3: Individual data points for both groups for event related desynchronization during grip termination from pre-intervention to post-intervention to follow up for the treatment group participants (solid lines) and the control group participants (segmented lines)

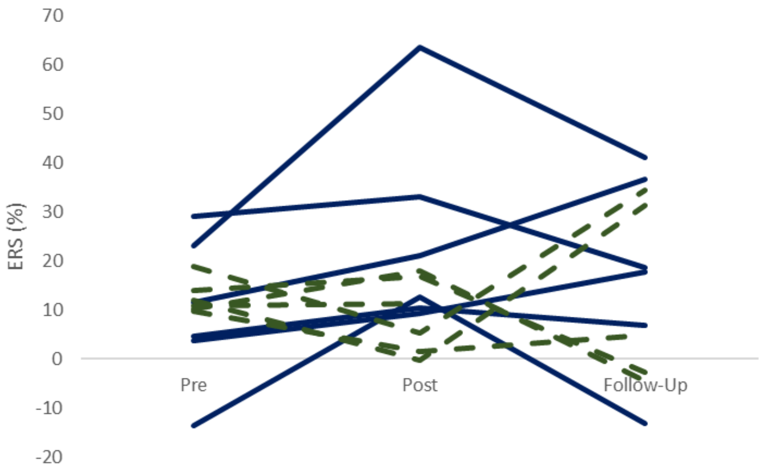

Supplementary Figure 4: Individual data points for both groups for event related resynchronization from pre-intervention to post-intervention to follow up for the treatment group participants (solid lines) and the control group participants (segmented lines)
